# The Association between Cognition and Academic Performance in Ugandan Children Surviving Malaria with Neurological Involvement

**DOI:** 10.1371/journal.pone.0055653

**Published:** 2013-02-01

**Authors:** Paul Bangirana, Jeremiah Menk, Chandy C. John, Michael J. Boivin, James S. Hodges

**Affiliations:** 1 Department of Psychiatry, Makerere University College of Health Sciences, Kampala, Uganda; 2 School of Public Health, University of Minnesota, Minneapolis, Minnesota, United States of America; 3 Department of Pediatrics, University of Minnesota, Minneapolis, Minnesota, United States of America; 4 Department of Psychiatry and Neurology/Ophthalmology, Michigan State University, East Lansing, Michigan, United States of America; 5 Neuropsychology Section, Department of Psychiatry, University of Michigan, Ann Arbor, Michigan, United States of America; INSERM U1094, University of Limoges School of Medicine, France

## Abstract

**Background:**

The contribution of different cognitive abilities to academic performance in children surviving cerebral insult can guide the choice of interventions to improve cognitive and academic outcomes. This study's objective was to identify which cognitive abilities are associated with academic performance in children after malaria with neurological involvement.

**Methods:**

62 Ugandan children with a history of malaria with neurological involvement were assessed for cognitive ability (working memory, reasoning, learning, visual spatial skills, attention) and academic performance (reading, spelling, arithmetic) three months after the illness. Linear regressions were fit for each academic score with the five cognitive outcomes entered as predictors. Adjusters in the analysis were age, sex, education, nutrition, and home environment. Exploratory factor analysis (EFA) and structural equation models (SEM) were used to determine the nature of the association between cognition and academic performance. Predictive residual sum of squares was used to determine which combination of cognitive scores was needed to predict academic performance.

**Results:**

In regressions of a single academic score on all five cognitive outcomes and adjusters, only Working Memory was associated with Reading (coefficient estimate = 0.36, 95% confidence interval = 0.10 to 0.63, p<0.01) and Spelling (0.46, 0.13 to 0.78, p<0.01), Visual Spatial Skills was associated with Arithmetic (0.15, 0.03 to 0.26, p<0.05), and Learning was associated with Reading (0.06, 0.00 to 0.11, p<0.05). One latent cognitive factor was identified using EFA. The SEM found a strong association between this latent cognitive ability and each academic performance measure (P<0.0001). Working memory, visual spatial ability and learning were the best predictors of academic performance.

**Conclusion:**

Academic performance is strongly associated with the latent variable labelled “cognitive ability” which captures most of the variation in the individual specific cognitive outcome measures. Working memory, visual spatial skills, and learning together stood out as the best combination to predict academic performance.

## Introduction

For many children, attainment of an education is associated with a better quality of life, longer life expectancy, and higher socioeconomic status [1], [2], [3]. Success in elementary subjects like reading and arithmetic depends on certain cognitive abilities. Working memory is needed when reading to decode unfamiliar words, retrieve semantic knowledge of familiar words, recall previously read text and anticipate where the passage is going [4]. Working memory is also used during arithmetic for multi-digit calculations and for activating, retrieving, and manipulating information in long-term memory [5], [6]. In performing calculations, visual spatial skills are also required to code the meaning of a digit in a multi-digit number [7], [8]. The child's attention capacity also aids in reading and is associated with academic performance [9], [10]. Deficient performance in any of these cognitive domains resulting from a cerebral insult is likely to affect reading and arithmetic thus compromising the child's education.

In sub-Saharan African, malaria with neurological impairment is a leading cause of impaired cognitive functioning [11]. Working memory and attention are common cognitive abilities that have been shown to be affected in prospective studies [12], [13]. A systematic review of studies of cognitive outcomes after malaria showed other deficits in language, executive functions, and visual spatial ability [14]. The possible effect of malarial infection on academic performance has been documented with lower grades observed in children who reported a higher number of malaria episodes [15]. Surprisingly, a recent study in Ugandan children did not find an effect of malaria with neurological impairment on reading, spelling, and arithmetic [16]. The authors attributed this to having assessed the children soon after the episode because cognitive effects after malaria are more pronounced several months after the illness [13].

Interventions to improve cognitive functioning in children after malaria have been tested with mixed results. In the first trial with Ugandan children, there was overall improvement in all cognitive and behavioural outcomes while in the second, minimal improvement was observed in learning only [17], [18]. In the second study, no effect was observed on reading, spelling, and arithmetic, which was attributed to the intervention not being tailored to improve academic outcomes.

Successful interventions for academic outcomes will need to train specific skills needed when performing reading, spelling, and arithmetic tasks. This requires identifying the key cognitive abilities associated with these academic performance outcomes and designing appropriate interventions. Identifying these cognitive abilities may also help predict possible academic difficulties after cerebral insult. The present study was designed to answer this question; specifically, it was designed to identify cognitive abilities that are associated with academic performance in children after malaria with neurological involvement.

## Methods

### Study population and recruitment

The present study was conducted between February 2008 and June 2010. The methods below have been described elsewhere [16]. Children were recruited from Mulago Hospital, the National Referral Hospital of Uganda, and from Nsambya, Rubaga, and Mengo Hospitals, all located in Kampala, Uganda's capital city. The latter three are large private mission hospitals. Participants were children aged 5 to 12 years presenting with malaria (*Plasmodium falciparum* on blood smears) and one or more of the following: 1) convulsive seizures lasting over 15 minutes or repeated seizures observed by the parent or during admission at the hospital; 2) impaired consciousness (Glasgow coma scale score of 14 and below); or 3) coma (i.e., unarousable coma with normal cerebrospinal fluid). A physical examination and medical history were done on admission. Exclusion criteria were history of or presence of meningitis, encephalitis, other central nervous system infections, sickle cell disease, epilepsy, multiple seizures, developmental delay, or hospitalization for malnutrition.

At discharge, home directions and telephone contacts were obtained from the parents/caregivers and an appointment made for the baseline assessment three months later. In the interim period, a home visit was made to assess the quality of the home environment.

Ninety children with malaria were recruited for the study. Of these, 10 did not meet the above inclusion criteria and were excluded and one died during admission, leaving 79 children for follow-up. By the three-month hospital visit, 17 children had either withdrawn from the study or had been lost to follow-up, leaving 62 children who were assessed for cognition and academic performance. The index conditions for these 62 children were cerebral malaria, malaria with seizures, and malaria with impaired consciousness, with 9, 34, and 19 children in each index, respectively. One of the 9 children with cerebral malaria had severe neurologic impairment from the disease and could not do the tests. The lowest possible cognitive test scores were assigned to this child.

Written informed consent was obtained from the parents or guardians of all study participants and, additionally, assent from children aged 7 years and older. Ethical approval for this study was granted by the Institutional Review Board for Human Studies at Makerere University College of Health Sciences and the Uganda National Council for Science and Technology.

### Assessments

#### Kaufman Assessment Battery for Children-second edition (KABC-II)

The KABC-II [19] is a comprehensive assessment of cognitive ability containing a number of critical scales that have been adapted, piloted, and validated in malaria studies among African children in Kenya [20], Senegal [21], and Uganda [12]. It retains its construct validity when used in Ugandan children to measure working memory, visual spatial ability, learning, and reasoning [22]. The measures used as predictors in the present study were working memory, visual spatial ability, learning, and reasoning.

#### Test of Variables of Attention (TOVA)

The TOVA is a computer administered visual continuous performance test used in diagnosis and monitoring of children and adults with attention deficit disorders [23]. The TOVA has been used in previous malaria studies in Senegal and Uganda [12], [13], [21]. It is a sensitive measure of cerebral insult from malaria as indicated by the persisting attention deficits at 24 months in Ugandan children with cerebral malaria [13]. The measure used as a predictor in the present study was the signal detection d′ prime score, a measure of overall attention capacity.

#### Wide Range Achievement Test-third edition (WRAT-3)

The WRAT-3 is a measure of reading, spelling, and arithmetic [24], [25]. It has been used earlier for research in Ugandan children with HIV [26]. Scores for reading, spelling, and arithmetic were used to determine the child's academic performance in those domains. Reading, spelling, and arithmetic were the primary outcomes.

#### Middle Childhood Home Observation for the Measurement of the Environment (MC-HOME)

The MC-HOME identifies parental behaviours that are important to a child's cognitive development and academic skills [27]. The MC-HOME as adapted to the Ugandan setting by Boivin and colleagues [12] was used in this study. It has 58 items measuring the amount of stimulation and learning opportunities available to the child in the home, which were summed into a single score. This score indicates the quality of the home environment, which is predictive of working memory performance in Ugandan children [3].

### Data analysis

Data was analysed using SPSS (version 17.0 Chicago, Il), M-Plus (version 6.1, Muthen & Muthen, Los Angeles, CA), and R (version 2.15.0, Vienna, Austria). Three different sets of linear regressions were run to examine the association between cognition and academic performance. In the first univariate regression, each of the three academic performance outcomes (reading, spelling, and arithmetic) was regressed on each of the five cognitive predictors (working memory, reasoning, learning, visual spatial skills, and attention) without any adjusters. In the second set, multivariate regressions were done with adjusters (age, sex, education level, nutritional status, and quality of the home environment) included in the regression model. In the third multivariate regression, each academic performance outcome was analyzed with all five cognitive measures included as predictors, along with the aforementioned adjusters.

An examination of potential latent factors was performed using an exploratory factor analysis (EFA) with an oblique GEOMIN rotation. One and two factor solutions were fit to the five factor indicators (working memory, reasoning, learning, visual spatial skills, and attention) and compared using the Bayesian Information Criterion (BIC). Next, a structural-equations model (SEM) was fit for each of the academic scores (reading, arithmetic, and spelling), relating the latent factor identified in the EFA with the academic performance score while controlling for age, sex, education level, nutritional status, and quality of the home environment. Last, the residual of each academic performance score, relative to the latent factor, was entered into the structural model in M-Plus using the MODINDICES option in the OUTPUT command. The statistical significance of including versus not including each residual path was determined by comparing the chi-square value from the MODINDICES output to a chi-square distribution with one degree of freedom. Standardized estimates of the paths relating the latent factors to each academic score are presented.

To consider which combination of cognitive tests best predicts academic performance, subsets of the five cognitive scores were compared according to their ability to predict, in a linear regression, the reading, spelling, and arithmetic performance measure for a new child. We used two criteria to compare subsets. The first was square-root (PRESS/N), where PRESS is the predictive residual sum of squares, i.e., the sum over children of the squared error in predicting a child's performance measure from a linear regression including all the other children [28]. Taking the square-root of this criterion divided by N gives an estimate of the standard deviation of prediction error in predicting a new child's performance measure. The second criterion was Mallows' Cp [28], which estimates the ratio of mean-squared prediction error divided by the regression equation's error variance, and includes a penalty for the number of predictors (cognitive scores) included. The purpose of using prediction-based methods to compare subsets is that they are less subject to problems arising from collinearity among the cognitive scores.

## Results

Most respondents were male (59.7%) with average age 7.22 years and an average 2.41 years in school (Table 1). When each of the three academic performance outcomes was regressed on each of the five cognitive scores, all regressions produced large coefficients with low p values (Table 2). Including the adjusters (age, sex, home environment, education level, and weight-for-age Z-score) resulted in lower coefficients (Table 2). Multiple regressions of a single academic score on all five cognitive outcomes and adjusters (Table 3), however, showed few significant associations. Working Memory was associated with Reading (coefficient estimate = 0.36, 95% confidence interval = 0.10 to 0.63, p = 0.009) and Spelling (0.46, 0.13 to 0.78, p = 0.008). Visual Spatial ability was associated with Arithmetic (0.15, 0.03 to 0.26, p = 0.014), and Learning was associated with Reading (0.06, 0.00 to 0.11, p = 0.049).

**Table 1:** Demographic characteristics of the children.

Variable	Range	M (SD)
Sex, male	37 (59.7)^1^	-
Age in years	5–12.25	7.22 (1.69)
HOME score	8–39	23.46 (6.22)
Years in school	0–8	2.41 (1.54)
Weight for age z-score	−3.22–2.05	−0.96 (1.14)

1 Results indicate frequency, N (%); HOME = Home observation for the measurement of the environment; N = Number; M = Mean; SD = Standard deviation.

**Table 2:** Regression coefficients with and without adjusters, excluding other cognitive measures.

		Reading		Spelling		Arithmetic	
		Slope	SE	Slope	SE	Slope	SE
Working memory	unadj*	0.94	0.10	0.86	0.10	0.72	0.08
	5 adj	0.54	0.09	0.54	0.11	0.45	0.08
Visual Spatial	unadj	0.50	0.06	0.41	0.06	0.44	0.04
	5 adj	0.26	0.06	0.22	0.07	0.27	0.05
Learning	unadj	0.20	0.02	0.17	0.02	0.15	0.01
	5 adj	0.11	0.02	0.11	0.03	0.10	0.02
Attention	unadj	2.78	0.66	2.31	0.64	2.50	0.50
	5 adj	1.50	0.44	1.23	0.54	1.38	0.38
Reasoning	unadj	1.25	0.20	1.19	0.19	1.02	0.16
	5 adj	0.36	0.18	0.55	0.20	0.40	0.16

* “unadj” is from the unadjusted analysis using a single cognitive scale; “5 adj” is from the analysis using a single cognitive scale adjusting for age, sex, home environment, education level, and weight-for-age z score.

**Table 3:** Regression coefficients with adjusters, including other cognitive measures.

	Academic achievement		
Predictors	Reading Coefficients (95% CI)	Spelling Coefficients (95% CI)	Arithmetic Coefficients (95% CI)
Working Memory	0.36 (0.10,0.63)**	0.46 (0.13,0.78)**	0.19 (−0.03,0.42)
Visual Spatial ability	0.07 (−0.06,0.20)	0.00 (−0.16,0.17)	0.15 (0.03, 0.26)*
Reasoning	0.01 (−0.29,0.31)	0.26 (−0.11,0.63)	0.11 (−0.15,0.37)
Learning	0.06 (0.00,0.11)*	0.06 (−0.01,0.13)	0.04 (−0.01,0.08)
Attention	−0.27 (−1.25,0.71)	−0.79 (−2.01,0.43)	−0.08 (−0.93,0.78)

* 0.01<P≤0.05;

** P≤0.01.

The low coefficients and large p-values when all five cognitive outcomes were entered in the model are due to the high correlations among the five cognitive measures (data not shown) suggesting a common underlying latent variable related to these five measures. A single factor was identified using EFA by comparing the factor solutions from the five predictors using BIC. Regressing the academic performance outcomes on the latent factor in an SEM that included the adjusters, a medium to large effect was found for each of the outcomes (Table 4; P<0.0001 for all three academic outcomes).

**Table 4:** Standardized estimates of regression coefficients on the ‘Cognitive ability’ latent variable.

Outcome	Estimate	S.E.	P-Value
Reading	0.717	0.094	<0.0001
Arithmetic	0.787	0.082	<0.0001
Spelling	0.699	0.105	<0.0001

The residuals, relative to the latent factor, of the five individual cognitive abilities were added as paths to the SEM separately, via M-Plus's modification indices, to test whether there was an additional association between the cognitive abilities and the performance outcome above and beyond that of the latent factor. No additional association was found above and beyond the latent cognitive ability (Table 5; P≥0.16 for all cognitive predictors and all academic outcomes).

**Table 5:** Estimates of regression coefficients of an outcome on a scale residual.

Reading	Estimate	StdYX Est	Mod Ind	P-value
Working Memory	0.172	0.180	1.015	0.31
Visual/Spatial	0.001	0.001	0.000	1.00
Reasoning	−0.109	−0.069	0.570	0.45
Learning	0.002	0.013	0.004	0.95
Attention	−0.407	−0.087	0.922	0.34
**Arithmetic**				
Working Memory	−0.007	−0.009	0.003	0.96
Visual/Spatial	0.090	0.200	1.775	0.18
Reasoning	−0.007	−0.005	0.003	0.96
Learning	−0.018	−0.114	0.303	0.58
Attention	−0.346	−0.090	0.904	0.34
**Spelling**				
Working Memory	0.201	0.207	0.975	0.32
Visual/Spatial	−0.055	−0.100	0.414	0.52
Reasoning	0.124	0.077	0.442	0.51
Learning	0.000	0.000	0.000	1.00
Attention	−0.746	−0.158	1.980	0.16

Given the above results and the difficulty and time taken administering each measure in this population of children, we examined whether it was necessary to measure all five cognitive scores. Specifically, we asked: When predicting a new child's reading, spelling, and arithmetic performance outcomes using these five cognitive scores (as well as the adjusters) in a linear regression, which subset of scores gives the best prediction? For this purpose, we defined “best prediction” using two criteria, predictive residual sum of squares (PRESS) and Mallows' Cp, which are described more fully in the Methods section. Table 6 shows results for PRESS, presenting the criterion square-root (PRESS/N) which estimates the standard deviation of prediction error in predicting a new child's academic performance outcomes; results for Mallows' Cp are similar and are available from the authors. For reading and spelling, all of the top 5 subsets of cognitive scores included working memory and all but two included learning. For arithmetic, all of the top 5 subsets of scores included visual/spatial, while 4 of 5 included learning and 3 of 5 included working memory. The other two cognitive scores, reasoning and attention, were included in only a few of the top-performing subsets of scores. Note also that the prediction-error criterion differs little for the top five subsets of scores in each group.

**Table 6:** Model selection of best predictors of academic performance using PRESS statistics.

Reading rank	Spelling rank	Arithmetic rank	WM	VS	REA	LEA	ATT	PRESS/N Reading
1	5	8	*			*		4.23
2	1	14	*			*	*	4.27
3	10	1	*	*		*		4.29
4	9	5	*	*		*	*	4.33
5	7	20	*		*	*		4.33

Spelling rank	Reading rank	Arithmetic rank	WM	VS	REA	LEA	ATT	PRESS/N Spelling
1	2	14	*			*	*	5.33
2	12	23	*		*			5.33
3	7	25	*		*	*	*	5.33
4	6	16	*					5.34
5	1	8	*			*		5.34

Arithmetic rank	Spelling rank	Reading rank	WM	VS	REA	LEA	ATT	PRESS/N Arithmetic
1	3	10	*	*		*		3.63
2	14	20		*		*		3.64
3	8	11	*	*				3.68
4	17	24		*		*	*	3.71
5	4	9	*	*		*	*	3.71

WM = working memory; VS = visual spatial skills; REA = reasoning; LEA = learning; ATT = attention.

## Discussion

The present study shows that in this group of children, who survived malaria with neurological involvement, reading, spelling, and arithmetic are strongly associated with a latent cognitive ability. This latent factor captures a large proportion of the variation in the five cognitive areas we tested. Individually, reading, spelling, and arithmetic were also associated with three of the five cognitive scores. Of the five scores, a combination of working memory, visual spatial skills, and learning was a better predictor of academic performance.

It has been shown that performance of classroom skills like reading, spelling, and arithmetic depends on certain cognitive functions [4], [7], [9]. Working memory especially is associated with all these functions as it is the ability to take up information and reproduce it after a short while. Our regression results regarding working memory's association with reading and spelling are in line with these findings. Similarly, visual skills are needed when solving arithmetic tasks, for example, following columns of numbers when doing sums [8]. The observed association between visual spatial ability and arithmetic supports this finding. The KABC-II learning score measures a child's ability to learn new items and retain them in long-term memory. This same measure was associated with reading ability in children. The association between working memory and visual spatial skills with academic performance was further shown using the predictive residual sum of squares criterion.

A large proportion of the five cognitive predictors is captured by a latent factor we called ‘cognitive ability’, with the residual (the unexplained part) of each predictor representing the unique component of that measure above and beyond the part of the measure captured in the latent factor. In the presence of this latent cognitive variable, none of the unique components of the individual measures was associated with any of the outcomes. All of the measures had relatively strong loadings on the factor, ranging from 0.68 to 0.90, with the latent factor accounting for 69% of the total variation for all of the measures. Thus none of the unique aspects of these cognitive measures appear to have a strong association with the academic outcomes.

In the regression analyses, the low association between working memory and academic outcomes could be a consequence of the heterogeneity within this predictor. The working memory outcome in this study is a summation of scores from the KABC-II subscales of Hand Movement (visual working memory), Number Recall (auditory working memory), and Word Order (visual/auditor working memory). Studies have shown that different components of working memory are associated with different arithmetic outcomes [29], [30]. Our study's working memory outcome thus did not measure a single construct of working memory, which may have weakened the association between it (and its residuals) and the academic performance outcomes as observed in both the SEM and regression analyses.

Also, different age groups of children have a preference for specific cognitive skills for learning. Younger children prefer visual strategies like pointing compared to older children who use verbal strategies like rehearsal [31]. Ugandan children in the same age range as these study children also have dominant cognitive abilities. Boivin and colleagues measured the association between visual, auditory and verbal working memory capacity in Senegalese and Uganda children [32]. Visual working memory predicted verbal working memory but this association was stronger for younger children (below 8.5 years). Auditory working memory also predicted verbal working memory capacity but this association was stronger for older children (above 8.5 years). In the present study, with an age range of 5 to 12 years, the dominance of different working memory modalities at different ages may have led to the low associations between working memory and academic performance outcomes.

This study found that as much as individual cognitive abilities are associated with academic outcomes, the latent factor from a latent variable approach that combined information from all of the cognitive measures had a stronger association with academic performance outcomes. The implication is that intervention studies aimed at improving academic performance in children by training cognitive abilities associated with schooling may have to target more than one cognitive ability. From the prediction analyses, the three likely candidates for training to improve academic performance are working memory, visual spatial skills, and learning which appear in the top predictive models for the academic scores.

Academic performance in this study was assessed using the WRAT-3, a measure of arithmetic, reading, and spelling. This method of standardised assessment ensured that all children were compared on the same measures. This is done on the assumption that study children from different schools have had the same quantitative and qualitative exposure in reading, spelling, and arithmetic which is unlikely. This omission of actual school results tailored to a particular school's syllabus and teaching competence is one limitation.

We therefore conclude that the variability in the different cognitive measures captured by a single latent variable is a better predictor of academic performance outcomes than the individual cognitive measures. A combination of working memory, visual spatial skills, and learning predicted academic performance better than all five cognitive scores together. Furthermore, none of the unique components of these cognitive measures, relative to the latent factor, were associated above and beyond the association found between the performance outcomes and the latent cognitive ability.

## References

[1] Lin RT, Chen YM, Chien LC, Chan CC (2012) Political and social determinants of life expectancy in less developed countries: a longitudinal study. BMC Public Health 12: 85. 22280469 10.1186/1471-2458-12-85 PMC3331806

[2] Forrest LF, Hodgson S, Parker L, Pearce MS (2011) The influence of childhood IQ and education on social mobility in the Newcastle Thousand Families birth cohort. BMC Public Health 11: 895. 22117779 10.1186/1471-2458-11-895 PMC3248886

[3] Bangirana P, John CC, Idro R, Opoka RO, Byarugaba J, et al (2009) Socioeconomic predictors of cognition in Ugandan children: implications for community interventions. PLoS One 4: e7898. 19936066 10.1371/journal.pone.0007898 PMC2774512

[4] Sesma HW, Mahone EM, Levine T, Eason SH, Cutting LE (2009) The contribution of executive skills to reading comprehension. Child Neuropsychol 15: 232–246. 18629674 10.1080/09297040802220029 PMC2728040

[5] McLean JF, Hitch GJ (1999) Working memory impairments in children with specific arithmetic learning difficulties. J Exp Child Psychol 74: 240–260. 10527556 10.1006/jecp.1999.2516

[6] Barrouillet P, Lepine R (2005) Working memory and children's use of retrieval to solve addition problems. J Exp Child Psychol 91: 183–204. 15925643 10.1016/j.jecp.2005.03.002

[7] Iglesias-Sarmiento V, Deano M (2011) Cognitive processing and mathematical achievement: a study with schoolchildren between fourth and sixth grade of primary education. J Learn Disabil 44: 570–583. 21444928 10.1177/0022219411400749

[8] de Hevia MD, Vallar G, Girelli L (2008) Visualizing numbers in the mind's eye: the role of visuo-spatial processes in numerical abilities. Neurosci Biobehav Rev 32: 1361–1372. 18584868 10.1016/j.neubiorev.2008.05.015

[9] Polderman TJC, Boomsma DI, Bartels M, Verhulst FC, Huizink AC (2010) A systematic review of prospective studies on attention problems and academic achievement. Acta Psychiatrica Scandinavica 122: 271–284. 20491715 10.1111/j.1600-0447.2010.01568.x

[10] Silva-Pereyra J, Bernal J, Rodriguez-Camacho M, Yanez G, Prieto-Corona B, et al (2010) Poor reading skills may involve a failure to focus attention. Neuroreport 21: 34–38. 19996811 10.1097/WNR.0b013e328332c566

[11] Idro R, Marsh K, John CC, Newton CR (2010) Cerebral malaria: mechanisms of brain injury and strategies for improved neurocognitive outcome. Pediatr Res 68: 267–274. 20606600 10.1203/PDR.0b013e3181eee738 PMC3056312

[12] Boivin MJ, Bangirana P, Byarugaba J, Opoka RO, Idro R, et al (2007) Cognitive impairment after cerebral malaria in children: a prospective study. Pediatrics 119: e360–366. 17224457 10.1542/peds.2006-2027 PMC2743741

[13] John CC, Bangirana P, Byarugaba J, Opoka RO, Idro R, et al (2008) Cerebral malaria in children is associated with long-term cognitive impairment. Pediatrics 122: e92–99. 18541616 10.1542/peds.2007-3709 PMC2607241

[14] Kihara M, Carter JA, Newton CR (2006) The effect of Plasmodium falciparum on cognition: a systematic review. Trop Med Int Health 11: 386–397. 16553922 10.1111/j.1365-3156.2006.01579.x

[15] Fernando D, Wickremasinghe R, Mendis KN, Wickremasinghe AR (2003) Cognitive performance at school entry of children living in malaria-endemic areas of Sri Lanka. Trans R Soc Trop Med Hyg 97: 161–165. 14584369 10.1016/s0035-9203(03)90107-6

[16] Bangirana P, Musisi S, Boivin MJ, Ehnvall A, John CC, et al (2011) Malaria with neurological involvement in Ugandan children: effect on cognitive ability, academic achievement and behaviour. Malar J 10: 334. 22047193 10.1186/1475-2875-10-334 PMC3225331

[17] Bangirana P, Allebeck P, Boivin MJ, John CC, Page C, et al (2011) Cognition, behaviour and academic skills after cognitive rehabilitation in Ugandan children surviving severe malaria: a randomised trial. BMC Neurology 11: 96. 21816079 10.1186/1471-2377-11-96 PMC3171314

[18] Bangirana P, Giordani B, John CC, Page C, Opoka RO, et al (2009) Immediate Neuropsychological and Behavioral Benefits of Computerized Cognitive Rehabilitation in Ugandan Pediatric Cerebral Malaria Survivors. J Dev Behav Pediatr 30: 310–318. 19668094 10.1097/DBP.0b013e3181b0f01b PMC2747354

[19] Kaufman AS, Kaufman NL (2004) Kaufman Assessment Battery for Children Manual. Circle Pines, MN: American Guidance Service.

[20] Holding PA, Stevenson J, Peshu N, Marsh K (1999) Cognitive sequelae of severe malaria with impaired consciousness. Trans R Soc Trop Med Hyg 93: 529–534. 10696414 10.1016/s0035-9203(99)90368-1

[21] Boivin MJ (2002) Effects of early cerebral malaria on cognitive ability in Senegalese children. J Dev Behav Pediatr 23: 353–364. 12394524 10.1097/00004703-200210000-00010

[22] Bangirana P, Musisi S, Allebeck P, Giordani B, John CC, et al (2009) A preliminary examination of the construct validity of the KABC-II in Ugandan children with a history of cerebral malaria. African Health Sciences 9: 188–192. PMC2887024 20589149

[23] Dupuy TR, Greenberg LM (2005) The T.O.V.A. Manual for IBM Personal Computer or IBM Compatible. Minneapolis, MN: Universal Attention Disorders.

[24] Wilkinson GS (1993) Wide-Range Achievement Test 3: Administration Manual. . Wilmington, Del.: Wide Range.

[25] Knoop A (2004) Test Review. Rehabilitation Counseling Bulletin 47: 184–185.

[26] Bagenda D, Nassali A, Kalyesubula I, Sherman B, Drotar D, et al (2006) Health, neurologic, and cognitive status of HIV-infected, long-surviving, and antiretroviral-naive Ugandan children. Pediatrics 117: 729–740. 16510653 10.1542/peds.2004-2699

[27] Caldwell BM, Bradley RH (2001) Home Inventory Administration Manual (3 rd ed.). Little Rock, AR: University of Arkansas.

[28] Weisberg S (1980) Applied Linear Regression. New York: Wiley.

[29] Simmons FR, Willis C, Adams AM (2012) Different components of working memory have different relationships with different mathematical skills. J Exp Child Psychol 111: 139–155. 22018889 10.1016/j.jecp.2011.08.011

[30] Zheng X, Swanson HL, Marcoulides GA (2011) Working memory components as predictors of children's mathematical word problem solving. J Exp Child Psychol 110: 481–498. 21782198 10.1016/j.jecp.2011.06.001

[31] Rasmussen C, Bisanz J (2005) Representation and working memory in early arithmetic. J Exp Child Psychol 91: 137–157. 15890174 10.1016/j.jecp.2005.01.004

[32] Boivin MJ, Bangirana P, Shaffer RC (2010) The Relationship between Visual-Spatial and Auditory-Verbal Working Memory Span in Senegalese and Ugandan Children. PLoS One 5: e8914. 20111706 10.1371/journal.pone.0008914 PMC2811730

